# OsQHB Improves Salt Tolerance by Scavenging Reactive Oxygen Species in Rice

**DOI:** 10.3389/fpls.2022.848891

**Published:** 2022-05-04

**Authors:** Jiahao Zhou, Jinzhu Qiao, Juan Wang, Ruidang Quan, Rongfeng Huang, Hua Qin

**Affiliations:** ^1^Biotechnology Research Institute, Chinese Academy of Agricultural Sciences, Beijing, China; ^2^National Key Facility of Crop Gene Resources and Genetic Improvement, Beijing, China

**Keywords:** OsQHB, salt stress, ROS scavenging, yield, rice

## Abstract

Soil salinity is a major environmental stress that restricts the growth and yield of crops. Mining the key genes involved in the balance of rice salt tolerance and yield will be extremely important for us to cultivate salt-tolerance rice varieties. In this study, we report a *WUSCHEL*-related homeobox (*WOX*) gene, quiescent-center-specific homeobox (*OsQHB*), positively regulates yield-related traits and negatively regulates salt tolerance in rice. Mutation in *OsQHB* led to a decrease in plant height, tiller number, panicle length, grain length and grain width, and an increase in salt tolerance. Transcriptome and qPCR analysis showed that reactive oxygen species (ROS) scavenging-related genes were regulated by OsQHB. Moreover, the *osqhb* mutants have higher ROS-scavenging enzymes activities and lower accumulation of ROS and malondialdehyde (MDA) under salt stress. Thus, our findings provide new insights into the role of rice *WOX* gene family in rice development and salt tolerance, and suggest that *OsQHB* is a valuable target for improving rice production in environments characterized by salt stress.

## Introduction

Soil salinity is one of the most widespread and significant soil problems worldwide, as salt hampers plant growth and development and hinders crop yield. As the primary source of food for more than half of the world population, rice is affected by salinity stress in varying degrees throughout all the developmental stages (Nam et al., 2015; Saini et al., 2018; He et al., 2019). Especially, rice is more sensitive to salinity during early seedling growth and flowering than other growth stages (Nam et al., 2015; Qin et al., 2019). Therefore, the growth ability of rice seedlings under salt stress conditions is considered an indicator of salt tolerance, and improving the salt tolerance of rice seedlings could increase the utilization of saline-alkali land and alleviate the world food crisis. Salt tolerance in rice is a polygenic trait controlled by quantitative trait loci (Ismail and Horie, 2017; Qin et al., 2020). Dissecting the key genes involved in rice salt tolerance and yield is an important objective to accelerating rice breeding.

Reactive oxygen species (ROS) is a versatile signal molecule that could be rapidly induced by a variety of environmental stresses (Miller et al., 2010; Lv et al., 2018). Generally, low concentrations of ROS function as signal molecules to regulate many biological processes, whereas high concentrations of ROS damage proteins, lipids, DNA, and carbohydrates (Miller et al., 2010; Xu et al., 2018; Yang and Guo, 2018). Therefore, maintaining an appropriate level of ROS is essential for plant growth and development. To avoid excessive accumulation of ROS and cause oxidative damage to cells, plants have evolved defense systems that include ROS-scavenging enzymes, such as superoxide dismutase (SOD), ascorbate peroxidase (APX), glutathione peroxidase (GPX), and catalase (CAT) (Foyer and Noctor, 2005). Various studies have shown that salt stress leads to excessive accumulation of ROS, and enhancing the activity of ROS-scavenging enzymes can improve the salt tolerance of plants (Lu Z. et al., 2007; Saini et al., 2018). For example, overexpression of *OsAPXa* or *OsAPXb* in *Arabidopsis* enhances tolerance to salinity stress (Lu Z. et al., 2007). Knockdown *GPX1* leads to enhanced photosynthesis impairment in response to salinity in rice (Lima-Melo et al., 2016). Transgenic rice plants overexpression *APX* exhibit reduced ROS accumulation and enhanced salt tolerance (Teixeira et al., 2006; Hong et al., 2007; Zhang et al., 2013).

The *WUSCHEL* related homeobox (*WOX*) gene family is one of plant homeobox (HB) transcription factor families (Yang et al., 2017). *WOX* genes have been shown to function in coordinate gene transcriptional related to shoot and root meristem establishment and organogenesis (Zhao et al., 2009; Hu and Xu, 2016; Cheng et al., 2018). In *Arabidopsis*, *AtWOX5* is specifically expressed in the quiescent center (QC) and acts as a key regulator of the root stem cell population (Sarkar et al., 2007; Forzani et al., 2014; Savina et al., 2020). AtWOX9 is essential to maintaining cell division and preventing premature differentiation in vegetative shoot apical meristem and early embryogenesis (Wu et al., 2005, 2007; Skylar et al., 2010). In rice, OsWOX3A modulates lateral root development and root hair formation through regulating auxin-transport gene expression (Yoo et al., 2013; Cho et al., 2016). OsWOX4 is involved in meristem maintenance and acts as a key regulator in early leaf development and primary root elongation (Ohmori et al., 2013; Yasui et al., 2018; Chen et al., 2020). OsWOX11 promotes crown root and shoot development by modulating cell proliferation in crown root meristem and shoot apical meristem (Zhao et al., 2009; Zhou et al., 2017; Cheng et al., 2018). Quiescent-center-specific homeobox (OsQHB), a homolog of AtWOX5, is involved in specification and maintenance of QC cell in root apical meristem and controlling lateral root primordium size (Kamiya et al., 2003; Kawai et al., 2022). All these studies suggest that *WOX* genes play an important role in regulating plant development.

As sessile organisms, plants must cope with various stresses in their environment to ensure the optimal combination of proliferation and survival. Accumulating studies show that *WOX* genes are associated with plant abiotic stress responses (Cheng et al., 2016; Liu H. et al., 2021; Wang L. Q. et al., 2021). In poplar, PagWOX11/12a positively regulates drought and salt tolerance by enhancing ROS scavenging capacity (Liu R. et al., 2021; Wang L. Q. et al., 2021). Knock-down of *GhWOX4* in cotton decreases the drought tolerance (Sajjad et al., 2021). In rice, overexpression of *OsWOX11* enhances drought tolerance by promoting root hair growth and development (Cheng et al., 2016). In addition, several rice *WOX* genes are responsive to salt stress (Cheng et al., 2014), implying that *WOX* genes might involve in improving the salt tolerance of rice. In the present study, we demonstrate that OsQHB coordinately regulates salt tolerance and yield of rice. Transcriptome analysis and physiological and biochemical indices show that OsQHB negatively regulates salt tolerance by improving ROS scavenging capacity. Thus, our researches enrich the functions of *WOX* genes in rice, and precisely manipulation of OsQHB could be useful to improve rice yield under salt stress.

## Results

### OsQHB Is a Nuclear Localization Protein and Mainly Expressed in Root and Shoot Apical Meristem

**FIGURE 1:**
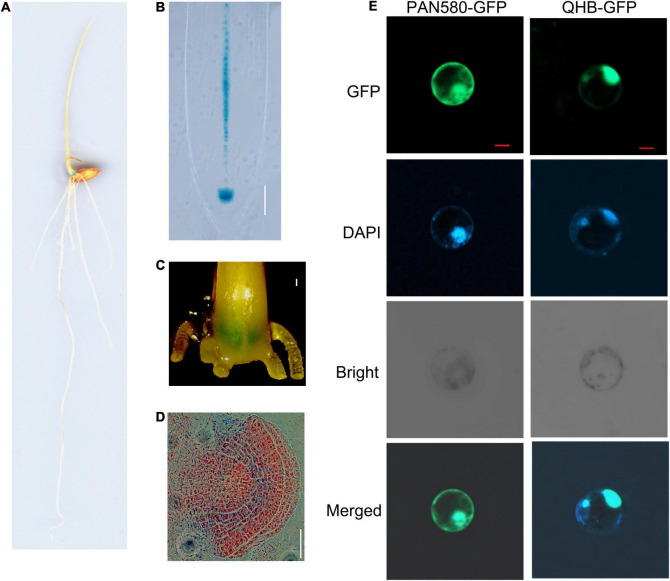
*OsQHB* expression and protein subcellular localization. **(A–D)** The tissue-specific expression of *OsQHB* revealed by promoter-GUS analysis. 3-d-old *OsQHBp-*GUS transgenic seedlings was used for GUS staining. At least 10 samples were observed for each organ and representative ones are presented. **(A)** 3-d-old seedling, **(B)** Primary root, bar = 100 μm, **(C)** Stem base, Bar = 200 μm. **(D)** crown root primordium. Bar = 200 μm. **(E)** Subcellular localization of OsQHB in rice protoplasts. Protoplasts were derived from etiolated shoots. Fluorescence from GFP and DAPI was detected by confocal microscopy. Merged indicates co-localization of OsQHB with DAPI. Bar = 5 μm. DAPI, 4′,6-diamidino-2-phenylindole.

WOX transcription factors play important roles in key developmental processes and in response to different abiotic stresses (Cheng et al., 2016; Jha et al., 2020; Wang L. Q. et al., 2021). Rice genome contains at least 13 *WOX* genes (Zhang et al., 2010). Among them, OsQHB encodes 200 amino acids including a homeodomain (HD) with 66 amino acids at the N-terminal (Supplementary Figure 1A). Phylogenetic analysis of rice and *Arabidopsis* WOX families indicated that OsQHB showed the highest similarity to AtWOX5 (Supplementary Figure 1B and Supplementary Table 1), which is specifically expression in the QC cells and is essential for stem cell maintenance in different meristems (Sarkar et al., 2007; Pi et al., 2015). β-glucuronidase (GUS) staining analysis using transgenic rice plants harboring *OsQHB* promoter-GUS construct showed that the transcription of *OsQHB* was detected in QC and stele of the root apexes, stem base, and crown root primordium (Figures 1A–D). Subsequently, the subcellular localization of OsQHB was investigated by fusing the OsQHB coding sequence with GFP and transiently expressed in rice protoplasts. The fluorescent signals of OsQHB-GFP fusion protein were found in the nucleus, as revealed by co-localization with the nuclear marker DAPI (4′,6-diamidino-2-phenylidone) (Yu et al., 2013; Figure 1E). These results suggest that OsQHB might be involved in the maintenance of the QC cells through a mechanism similar to that of AtWOX5.

### OsQHB Positively Regulates Yield-Related Traits

To study the function of OsQHB, we generated knockout mutants in the Nipponbare (NIP) background *via* CRISPR-Cas9 mediated genome editing and isolated three alleles for further investigation. The *osqhb-1* and *osqhb-2* mutant plants contained a 5-bp and 8-bp deletion, whereas the *osqhb-3* mutant plants contained a 1-bp insertion, in the coding region of the target gene, correspondingly, leading to a frame shift in the open reading frame and the generation of a premature stop codon (Supplementary Figures 2A,B). We also generated overexpression (OE) lines containing the coding region of *OsQHB* under the control of the CaMV35S promoter, the increased expression of the target gene was confirmed by qPCR (Supplementary Figure 2C). Although the expression of *OsQHB* was significantly increased in *OsQHB-OE* plants, whereas the OsQHB protein was increased slightly in *OsQHB-OE* plants (Supplementary Figure 2D), suggesting that OsQHB may be rapidly degraded by the ubiquitin system or the proteasome. To verify this hypothesis, we treated *OsQHB-OE* plants with MG132 (an inhibitor of the ubiquitin proteasome system) and then detected the OsQHB protein levels. The results showed that MG132 treatment significantly increased the accumulation of OsQHB protein (Supplementary Figure 2D), indicating that OsQHB is rapidly degraded by the ubiquitin proteasome system, and implying that OsQHB is essential for plant development and plants must maintain low levels of OsQHB protein to ensure normal growth.

**FIGURE 2:**
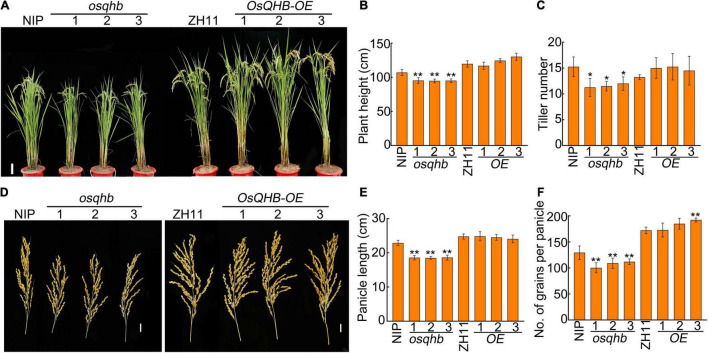
Mutation in *OsQHB* reduced plant height, tiller number, panicle length, and number of grains per panicle. **(A)** Phenotypic comparison of field-grown wild-type and *OsQHB* transgenic plants. Bar = 10 cm. **(B,C)** Plant height **(B)** and tiller number **(C)** of field-grown wild-type and *OsQHB* transgenic plants. Values are means ± SD (*n* ≥ 20). * and ** indicates significant difference compared to NIP at *P* < 0.05 and *P* < 0.01 by Student’s *t*-test. **(D)** Panicle phenotypes of wild-type and *OsQHB* transgenic plants. Bar = 2 cm. **(E,F)** Panicle length **(E)** and number of grains per panicle **(F)** of wild-type and *OsQHB* transgenic plants. Each value is average of 20 plants. Bars indicate SD. ** indicates significant difference compared to NIP or ZH11 at *P* < 0.01 by Student’s *t*-test.

Next, we investigated the agronomic traits of *OsQHB* overexpressed plants and *osqhb* mutants. At maturation stage, the *osqhb* mutants were shorter and the *OsQHB-OE* plants were slightly taller than wild-type plants (Figures 2A,B). The number of effective tiller and panicle length in wild-type and *OsQHB-OE* plants were identical, whereas *osqhb* mutants produced less effective tiller and smaller panicle than the wild type (Figures 2C–E). The total number of grains per panicle was significantly reduced in *osqhb* mutants, whereas slightly increased in *OsQHB-OE* plants, compared to that of the wild type (Figure 2F). All these analyses indicate that modulation of *OsQHB* expression affects yield-related traits in rice.

**FIGURE 3:**
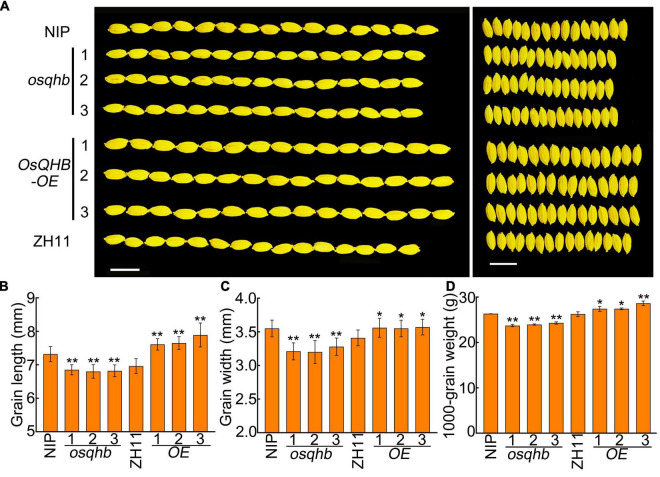
OsQHB positively regulates grain size. **(A)** Phenotypic comparison of well-filled grains of wild-type and *OsQHB* transgenic plants. Bar = 1 cm. **(B–D)** Grain length **(B)**, grain width **(C)** and 1,000-grain weight **(D)** from well-filled grains. Values are means ± SD of three replicates. * and ** indicates significant difference compared to NIP or ZH11 at *P* < 0.05 and *P* < 0.01 by Student’s *t*-test.

The grain size was also examined in *osqhb* mutants and *OsQHB-OE* plants using well-filled grains. The grain length and grain width was significantly reduced in *osqhb* mutants, whereas increased in *OsQHB-OE* plants, compared with those in the wild type (Figures 3A–C). The thousand-grain weight of various plants was further measured. The results showed that *osqhb* mutants had significant reductions, whereas the *OsQHB-OE* plants had significant increases in this parameter (Figure 3D). These results indicate that OsQHB positively regulates the grain size and thousand-grain weight in rice, implying that manipulation of OsQHB could be useful to improve rice yield.

### *OsQHB* Is Involved in Abiotic Stress and Hormone Response

**FIGURE 4:**
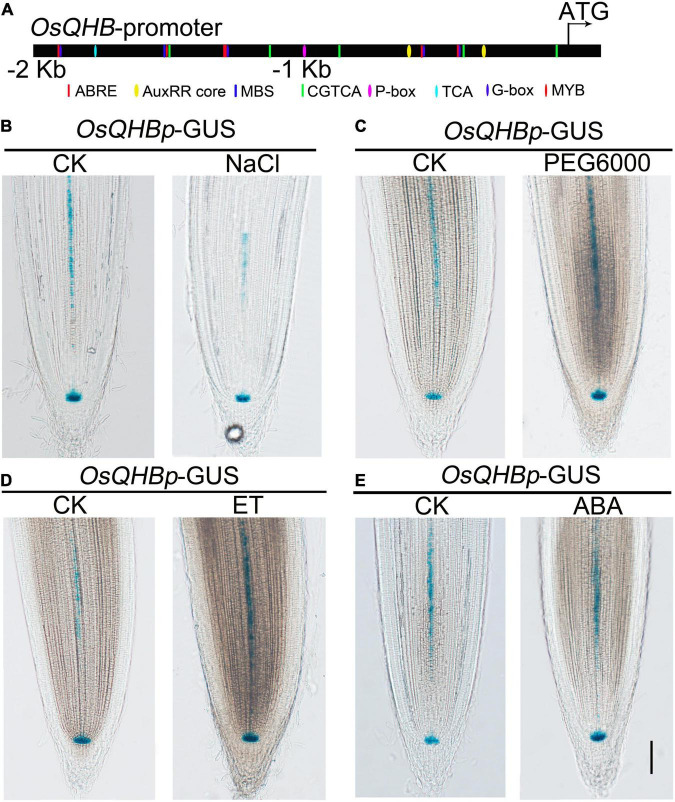
Expression of *OsQHB* under stress and hormone treatments. **(A)** Distribution of major stress-related *cis-*elements in the promoter region of *OsQHB*. **(B–E)** GUS staining of 3-d-old transgenic lines contained *OsQHBp*-GUS treated with 100 mM NaCl **(B)**, 10% PEG6000 **(C)**, 10 μL/L Ethylene **(D)**, or 1 μm ABA **(E)** treatment. Bar = 100 μm.

The promoter sequence of *OsQHB* contains many putative hormone- and stress-response *cis-*elements, such as ABRE element (4 hits), AuxRR core (2 hits), P-box (1 hit), G-box (6 hits), and MYB recognition site (1 hit) (Figure 4A). ABRE element is associated with ABA response (Nakashima and Yamaguchi-Shinozaki, 2013), G-box and MYB recognition site with drought response (Ezer et al., 2017), AuxRR core with auxin response, and P-box with gibberellin response (Sakai et al., 1996; Hwang et al., 2010; Wang Y. et al., 2011; Ambawat et al., 2013; Wei et al., 2013; Zhang et al., 2020; Dutta et al., 2021). These elements are contained in the promoter of *OsQHB* suggests that OsQHB might be involved in modulating hormone response and stress tolerance.

Furthermore, we examined the responses of *OsQHB* to various abiotic stresses and hormone treatments by β-glucuronidase (GUS) staining. Considering that *OsQHB* is mainly expressed in root tips, we selected root tips for GUS staining. Our results showed that the GUS staining was significantly repressed by NaCl and weakly induced by osmotic stress (PEG6000) and ethylene, but apparently not affected by abscisic acid (ABA) treatment, respectively (Figures 4B–E). Numerous studies have shown that ABA and ethylene play important roles in regulating salt and drought tolerance (Pan et al., 2012; Liang et al., 2019; Liu C. Y. et al., 2019; Luo et al., 2020). Collectively, these results indicate that OsQHB might be associated with ethylene- and ABA-related environmental stimuli in rice.

### OsQHB Negatively Regulates Salt Tolerance Through Modulating ROS Scavenging

**FIGURE 5:**
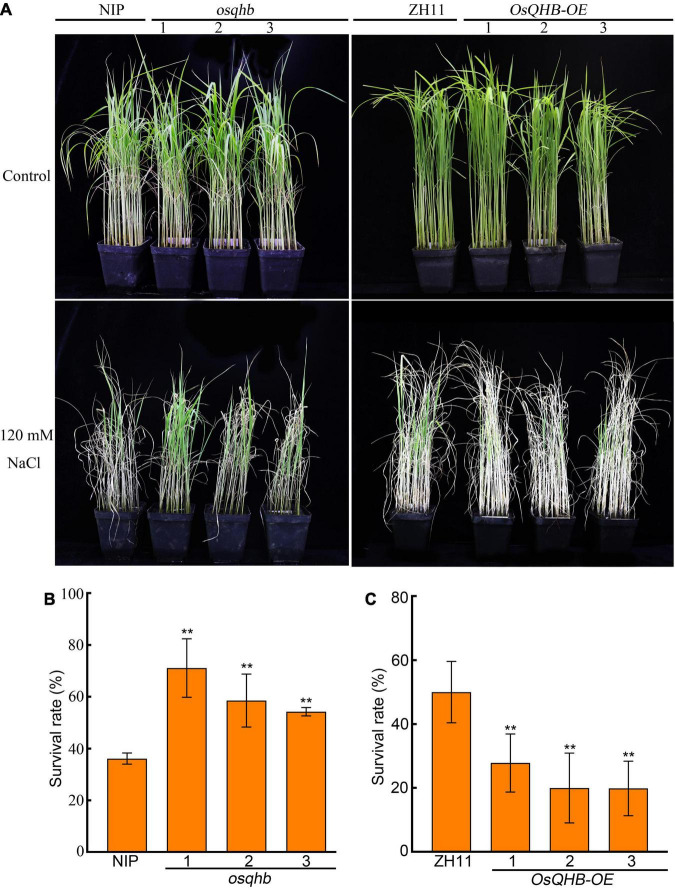
OsQHB negatively regulates salt tolerance of rice. **(A)** Phenotypes of wild-type and *OsQHB* transgenic plants under salt stress. 3-week-old wild-type and *OsQHB* transgenic plants were treated with or without 120 mM NaCl for 10 days. **(B,C)** Survival rates of the plants shown in panel **(A)** after recovery for 10 days. Approximately 50–60 seedlings were used per experiment. Data are the mean ± SD of 3 biological replicates. ** indicates significant difference compared to NIP or ZH11 at *P* < 0.01 by Student’s *t*-test.

Salt stress is a major environmental problem globally by affecting plant growth and causing crop production. Increasing numbers of studies have shown that the *WOX* gene family plays a role in salt tolerance (Wang L. Q. et al., 2021). As salt represses *OsQHB* transcription, we hypothesized that OsQHB is involved in regulating salt tolerance in rice. To verify this, we treated wild type, *osqhb*, and *OsQHB-OE* seedlings with NaCl. After 10 days of treatment with 120 mM NaCl, *osqhb* seedlings were obviously less affected than NIP seedlings by salt stress, whereas *OsQHB-OE* seedlings were all wilted to death (Figure 5A). After recovery for 10 days in non-salt conditions, the survival rate of *osqhb* seedlings was significantly higher, and that of *OsQHB-OE* seedlings was significantly lower, than that of the wild-type seedlings (Figures 5B,C). These results indicate that OsQHB negatively regulates the response of rice to salt stress at seedling stage.

**FIGURE 6:**
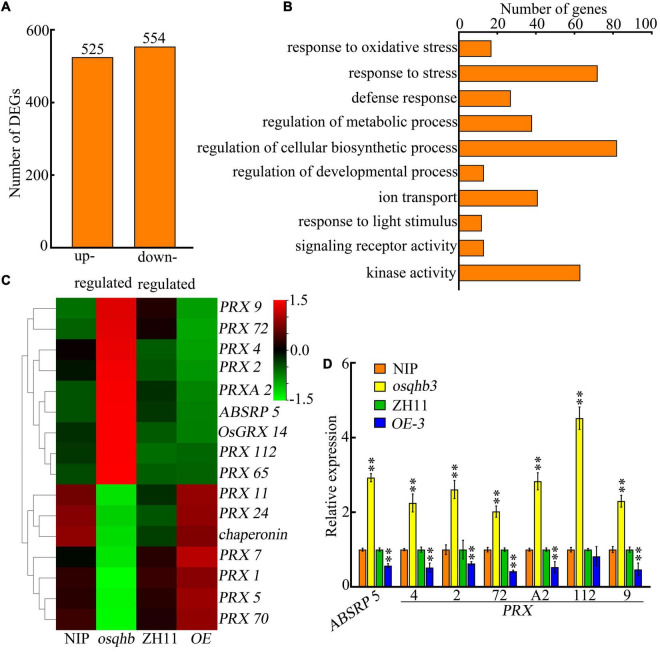
Transcriptome analysis reveals differentially expressed genes (DEGs) in *OsQHB* transgenic plants. **(A)** Number of DEGs up- and down-regulated in *osqhb* mutants. **(B)** Gene ontology (GO) analysis of *OsQHB*-regulated genes. **(C)** Heat map of the microarray expression profiles of ROS scavenging genes in *OsQHB* transgenic plants. **(D)** Quantitative real-time PCR (qPCR) analysis of ROS scavenging genes in 3-d-old wild-type and *OsQHB* transgenic seedlings. *OsActin1* was used as an internal control. Three biological replicates were performed with similar results, and a representative experiment is shown. Samples were collected from three independent experiments. Bars indicate ± SD. The asterisks indicate significant differences compared to NIP or ZH11 (^**^P < 0.01, Student’s *t*-test).

To elucidate the molecular mechanisms regulated by OsQHB, we compared the transcriptomes of wild type, *osqhb*, and *OsQHB-OE* seedlings using transcriptome deep sequencing (RNA-seq). Totally 1079 differential expressed genes (DEGs) were identified in *osqhb* mutants, including 525 up-regulated DEGs and 554 down-regulated DEGs (Figure 6A and Supplementary Table 2). Gene Ontology (GO) enrichment analysis showed that these OsQHB-regulated DEGs were involved in stress response, regulation of metabolic and cellular biosynthetic process, ion transport, and kinase activity (Figure 6B), indicating that OsQHB is involved in a variety of biological processes and molecular functions, including those associated with abiotic stress responses.

Previous studies have shown that salt stress leads to over-production of ROS, and enhanced ROS scavenging capacity can improve salt tolerance in plants (Steffens, 2014; Xu et al., 2018; Yang and Guo, 2018). Thus we analyzed the expression of ROS scavenging-related genes in transcriptome data and found that multiple peroxidase (*PRX*) genes were regulated by OsQHB (Figure 6C and Supplementary Table 2), this regulation was further confirmed by qPCR (Figure 6D). *PRX* genes play diverse roles in plant physiology by scavenging ROS (Liu R. et al., 2021). These results indicate that OsQHB regulates the expression of ROS scavenging-related genes, which could further affect ROS accumulation.

**FIGURE 7:**
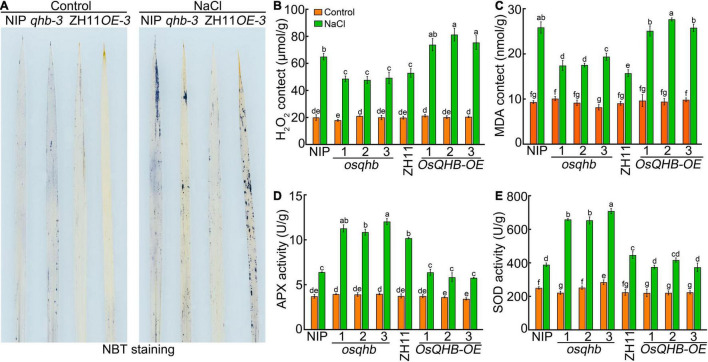
Loss of OsQHB function enhanced the ROS scavenging capability under salt stress. **(A)** NBT staining of 3-week-old wild-type and *OsQHB* transgenic plant leaves treated with or without 120 mM NaCl treatment for 3 days. **(B,C)** Hydrogen peroxide (H_2_O_2_) **(B)** and malondialdehyde (MDA) **(C)** contents in leaves of 3-week-old wild-type and *OsQHB* transgenic plants treated with or without 120 mM NaCl treatment for 3 days. **(D,E)** Ascorbate peroxidase (APX) **(D)** and Superoxide dismutase (SOD) **(E)** activities in leaves of 3-week-old wild-type and *OsQHB* transgenic plants treated with or without 120 mM NaCl treatment for 3 days. For **(B)** to panel **(E)**, the data are shown as mean ± SD. *n* = 3 biological replicates. Statistically significant differences are indicated by different letters in panels **(B–E)** (*P* < 0.05, one-way ANOVA with Tukey’s test).

To investigate whether ROS levels were altered in wild type, *osqhb*, and *OsQHB-OE* seedlings under salt treatment, we measured ROS levels in wild type, *osqhb*, and *OsQHB-OE* seedling leaf. Nitro blue tetrazolium (NBT) staining showed that the *osqhb* mutants accumulated less O^2–^ in the leaves compared with wild-type plants under salt stress (Figure 7A). On the contrary, the *OsQHB-OE* plants accumulated much more O^2–^ in the leaves compared with wild-type plants under salt stress (Figure 7A). Further determination of H_2_O_2_ content showed that the H_2_O_2_ content of *osqhb* mutants was lower, while that of *OsQHB-OE* plants was higher, than that of wild type after salt treatment (Figure 7B). Malondialdehyde (MDA) is an important product of lipid peroxidation, which represents the degree of oxidative damage to the plant cell (Qin et al., 2016). Salt stress significantly increased the MDA content in wild-type (Figure 7C). This tendency was weakened in *osqhb* mutants, but enhanced in *OsQHB-OE* plants (Figure 7C). These results indicate that knocking out *OsQHB* leads to the reduction of ROS accumulation under salt stress, thereby improving the salt tolerance of rice seedlings.

Salt tolerance is tightly related to the activities of ROS-scavenging enzymes. Therefore, we measured the activities of ROS-scavenging enzymes after exposed to NaCl. As shown in Figures 7D,E, after exposed to salinity stress, the activities of antioxidant enzymes in all the three genotypes were significantly increased, whereas the *osqhb* mutants showed a higher antioxidant enzymes activities and the *OsQHB-OE* lines showed a lower antioxidant enzymes activities, as compared with wild-type plants (Figures 7D,E), indicating that the *OsQHB* mutation leads to enhancement of salt tolerance in rice seedlings by enhancing the activities of antioxidant enzymes.

## Discussion

Maintaining stable, high yields under fluctuating environmental conditions is a long-standing goal of crop improvement. However, due to internal trade-off mechanisms in plants, high yields and high biotic/abiotic resistance are usually incompatible (Deng et al., 2017). Therefore, dissecting genes that simultaneously regulate grain size and salt resistance is a promising strategy for improving both grain yield and salt tolerance in crop. In the present study, we demonstrated that OsQHB positively regulates yield-related traits but negatively regulates salt tolerance in rice. Further investigations showed that OsQHB regulates salt tolerance by enhancing ROS scavenging capacity. Our findings deepen our understanding of the functions of rice *WOX* gene family in response to salt stress and provide useful genes for improving yield and salt tolerance in rice.

WOX is a transcription factor family that plays a critical role in plant growth and development (Zhao et al., 2009; Hu and Xu, 2016; Chen et al., 2020). Accumulating studies show that *WOX* genes are associated with plant abiotic stress responses (Cheng et al., 2016; Liu H. et al., 2021; Wang L. Q. et al., 2021). In poplar, PagWOX11/12a positively regulates drought resistance through modulating ROS scavenging (Liu R. et al., 2021). Here, we showed that mutation in *OsQHB* weakened the accumulation of ROS under salt stress. Correspondingly, the *osqhb* mutants exhibited higher ROS-scavenging enzyme activity after salt treatment. Transcriptome and qPCR analysis showed that ROS scavenging-related genes were regulated by OsQHB, indicating that OsQHB improves rice salt tolerance by enhancing ROS scavenging capability through possibly regulating ROS scavenging-related genes expression. These findings deepen our understanding of the role of *WOX* genes in salt resistance. Further investigations will be required to determine how ROS scavenging-related genes are regulated by OsQHB to enhance salt tolerance.

The root system architecture of crops can affect their production, particularly in abiotic stress conditions (Uga et al., 2013; Kitomi et al., 2020). Numerous studies have shown that *WOX* gene family improves crop resistance to abiotic stress by modulating root system architecture (Cheng et al., 2016; Wang L. Q. et al., 2020). In *Arabidopsis*, *AtWOX5* is specifically expressed in QC and acts as a root stem cell organizer (Sarkar et al., 2007). Loss of *AtWOX5* function in the root stem cell niche causes terminal differentiation of columella stem cells (Sarkar et al., 2007). OsQHB is a homolog of AtWOX5, suggesting that OsQHB and AtWOX5 may have similar functions. Previous studies have shown that *OsQHB* is involved in the specification and maintenance of QC cells in the root apical meristem, and overexpression of *OsQHB* leads to abnormal root formation (Kamiya et al., 2003). In this study, our results show that OsQHB is localized in the nuclear and specifically expressed in QC cells of the root and stem base, which is consistent with previous reports (Kamiya et al., 2003). Stem base is where the crown root initiates (Zhao et al., 2009), and our results show that *OsQHB* is also expressed at the crown root primordial, indicating the important role of OsQHB in rice root development. Moreover, OsQHB negatively regulates salt tolerance in rice seedlings by enhancing ROS scavenging capability, suggesting that OsQHB could be useful for the controlled breeding of root system architectures that are adapted to the salinity conditions. Subsequent studies should focus on dissecting the function and regulatory network of OsQHB in root development.

Rice is considered to be a salt-sensitive species and salt-activated inhibition of plant growth may represent a positive mechanism to help plants adapt to salinity stress (Chinnusamy et al., 2005; Achard et al., 2006; Wang J. et al., 2020), this means that the development of superior crop cultivars with both high yields and high abiotic stress resistance is a tremendous challenge for crop breeding. Identifying key genes involved in the balance of rice salt tolerance and yield and discovering favorable alleles for these genes could be used to enhance both yield and abiotic stress resistance in rice. In this study, our results showed that knocking out *OsQHB* enhances the salt tolerance of rice seedling by improving ROS scavenging capability. Moreover, mutation in *OsQHB* significantly reduced yield-related traits, whereas overexpression of *OsQHB* had no obvious effect, likely due to rapidly degradation of OsQHB protein by the ubiquitin proteasome system, as revealed by MG132 treatment. Therefore, precisely manipulation of OsQHB represents a promising strategy for improving both grain yield and stress tolerance in rice. Further study should focus on mining favorable alleles of OsQHB and elucidating the molecular mechanism by which OsQHB is degraded.

Taken together, our research presents new insights into the roles of OsQHB in improving yield and salt tolerance in rice, and dissecting the underlying mechanism and mining favorable alleles of OsQHB will facilitate the practical use of OsQHB in breeding superior crop cultivars with both high yields and high salt resistance.

## Materials and Methods

### Plant Material and Growth Conditions

*OsQHBp-GUS* transgenic line was described previously (Ni et al., 2014). *osqhb* allelic mutants in the Nipponbare (NIP) background were generated by using CRISPR/Cas9 (Xu et al., 2017). Briefly, the target region 5′-GGAGCAGGTGAAGGTCCTGA-3′ was introduced into the pHUN4c12 vector backbone, and the recombinant vector was transformed into *Agrobacterium* strain EHA105-pSOUP for rice transformation. To generate the overexpression transgenic plants, the coding sequence of *OsQHB* was amplified by PCR and cloned in-frame with a Flag-tag into pCAMBIA1307 under the control of the *CaMV 35S* promoter (Wang J. et al., 2013). The recombinant plasmids were introduced separately into NIP or Zhonghua 11 (ZH11) *via* Agrobacterium-mediated transformation.

Rice seeds were imbibed in Petri dishes with sterile distill water at 37°C for 48 h. The germinated seeds were sown in a bottomless 96-well plate in a container of Yoshida’s culture solution (Cui et al., 2015) or in growing trays filled with soil. The plants were grown in a growth chamber under a 14 h light (30°C)/10 h dark (25°C) photoperiod, with a light intensity of ∼150 μmol m^–2^s^–1^ (white light) and 60% relative humidity. To assess salt-tolerance of rice seedlings, 3-week-old seedlings were treated with 120 mM NaCl solution for 10 days, and then transferred to water without NaCl for an additional 10 days. Subsequently, the survival rate of rice seedlings was counted (surviving seedlings indicated plants with green leaves).

For propagation and investigation of agronomic traits, the plants were cultivated at the Experimental Station of the Chinese Academy of Agricultural Sciences in Beijing during the natural growing seasons.

### Phylogenetic and Promotor Analysis

*Arabidopsis* WOX proteins sequences were available at the *Arabidopsis* genome sequence database^1^ and rice WOX proteins sequences were downloaded from the National Center for Biotechnology Information (NCBI) database^2^. Full-length protein sequences of all WOX proteins were aligned by multiple sequence alignment program of the Clustal X, and then phylogenetic tree was constructed by MEGA 6 software according to the following parameters: neighbor-joining method, 500 bootstraps (Tamura et al., 2007).

For promoter analysis, the promotor sequence (2,000 bp upstream of ATG) of *OsQHB* was uploaded to the PlantCARE database^3^ to identify stress related *cis-*elements as previously reported (Yang and Guo, 2018).

### Quantitative Real-Time PCR and β-Glucuronidase Staining

Total RNA was extracted from leaf tissues using an Ultrapure RNA Kit (CWBIO, China) according to the manufacturer’s instructions. ∼2 μg total RNA was reverse transcribed using HiScript II Q RT SuperMix (Vazyme, China) according to the manufacturer’s instructions. qPCR was performed on an optical 96-well plate with Bio-Rad iQ5 system using SYBR Green Real-time PCR Master Mix reagent (Vazyme, China) as previously described (Zhang et al., 2012). The rice *Actin1* gene was used as an internal standard to normalize gene expression. The primers used for qPCR are listed in Supplementary Table 3.

For GUS staining, 3-d-old *OsQHBp-GUS* seedlings were immersed in GUS staining buffer (50 mM sodium phosphate, pH 7.0; 10 mM EDTA; 0.5 mM K_3_[Fe (CN)_6_]; 0.5 mM K_4_[Fe (CN)_6_]; 0.1% [v/v] Triton X-100; and 1 mM 5-bromo-4-chloro-3-indolyl-β-D-glucuronic acid). The samples were vacuumed in a vacuum pump for 30 min, and then stained in 37°C for 2 d. After dehydrated in 75% (v/v) ethanol to remove the chlorophyll, the root tip was sectioned and photographed under a stereoscopic microscope.

### Subcellular Location

To determine the subcellular localization of OsQHB, the full coding sequence of *OsQHB* was cloned into the pAN580-GFP vector under the control of the *CaMV 35S* promoter. Rice protoplasts prepared from etiolated shoots were transfected with OsQHB-GFP (Jiang et al., 2018). After incubated at 28°C for 16–20 h, equal volumes of DAPI (4′,6-diamidino-2-phenylidone) staining solution and protoplast suspension were mixed, and the fluorescence signal was observed with a confocal laser scanning microscope.

### Nitro Blue Tetrazolium Assay for the Superoxide Anion

The leaves of 3-week-old seedlings were immersed in 20 mM phosphate buffer (pH 6.1) containing 2 mM NBT. After vacuumed in pump for 1 h, the samples were stained in 37°C for 12 h and then transferred to 75% (v/v) ethanol to stop the reaction. The material was photographed under a light stereomicroscope.

### Superoxide Dismutase and Ascorbate Peroxidase Activity Measurement

Ascorbate peroxidase and SOD activities were determined according to the instruction of the superoxide dismutase assays kits (Solarbio, China) and the ascorbate peroxidase assays kits (Solarbio, China). In brief, 0.1 g of normal or salt-stressed rice leaves from 3-week-old seedlings were harvested, ground in liquid nitrogen, and extracted by extraction buffer. After centrifuged at 8,000 *g* (SOD) for 10 min or 13,000 *g* (APX) for 20 min, the supernatant was used for SOD and APX activity measurement. The absorbance values were measured at 560 and 290 nm, respectively. SOD and APX activities were calculated from the formula provided with the SOD and APX assay kit (Solarbio, China).

### Measurement of Hydrogen Peroxide and Malondialdehyde Contents

H_2_O_2_ content was determined using the method described previously (Wang Y. et al., 2019). Briefly, 0.1 g rice leaves from 3-week-old seedlings with or without salt treatment were harvested and homogenized in 1 ml cold acetone. Then, H_2_O_2_ content was determined using a hydrogen peroxide assay kit (Solarbio, China) according to the manufacturer’s instructions.

To measure MDA, 0.1 g of normal or salt-stressed rice leaves were homogenized in 1 ml 0.1% (w/v) trichloroacetic acid (TCA) followed by centrifugation at 8,000 *g* for 10 min at 4°C. Four volumes of 0.5% (w/v) thiobarbituric acid (TBA) in 20% (w/v) TCA were added to one volume of supernatant; the mixture was incubated at 100°C for 1 h. The reaction was terminated by incubating the mixture on ice for 15 min, and the absorbance was measured by spectrophotometry at 450 nm, 532 and 600 nm. The content of MDA was calculated according to the formula provided in the MDA assay kit (Solarbio, China).

### Transcriptome Analysis

The primary roots of 3-d-old *OsQHB* overexpression line, *osqhb* mutant, and wild-type seedlings were collected and extracted using the TRIzol method (TIANGEN BIOTECH, China) and treated with RNase-free DNase I (TaKaRa, Japan). mRNA was purified from total RNA using poly-T oligo-attached magnetic beads and then subjected to RNA-seq library construction for the transcriptome experiments using NEBNext^®^ Ultra™ RNA Library Prep Kit for Illumina^®^ (NEB, United States). Multiplex paired-end adapters were used for multiplex libraries. The library quality was assessed on the Agilent Bioanalyzer 2100 system. The library preparations were sequenced on an Illumina Hiseq 4000 platform by the Beijing Allwegene Technology Company Limited (Beijing, China) and paired-end 150 bp reads were generated. After removing adaptor and low-quality reads, clean reads were mapped to rice genome MSU7.0 using TopHat, and analyzed using Cufflinks according to Trapnell et al. (2012). Poisson-dispersion model of fragment was used to conduct statistical analysis (FDR < 0.05) and responsive genes were identified by fragments per kilobase per million reads (FPKM) requiring more than twofold change between two samples. Three biological replicates were used, and their repeatability and correlation were evaluated by the Pearson’s Correlation Coefficient (Schulze et al., 2012). The transcriptome data from this study can be found in the National Center for Biotechnology Information Sequence Read Archive (NCBI SRA) under the accession number PRJNA801123.

### Primer Sequences

The primers used in this study are shown in Supplementary Table 3.

## Data Availability Statement

The datasets presented in this study can be found in online repositories. The names of the repository/repositories and accession number(s) can be found below: National Center for Biotechnology Information Sequence Read Archive (NCBI SRA) under the accession number PRJNA801123.

## Author Contributions

JZ: performed the research, data curation and writing-original draft. JQ: performed the research. RQ: analyzed and discussed the data. JW: analyzed and discussed the data. RH: project administration, writing-review and editing. HQ: conceptualization, supervision, designed, and writing-review and editing. All authors contributed to the article and approved the submitted version.

## Conflict of Interest

The authors declare that the research was conducted in the absence of any commercial or financial relationships that could be construed as a potential conflict of interest.

## Publisher’s Note

All claims expressed in this article are solely those of the authors and do not necessarily represent those of their affiliated organizations, or those of the publisher, the editors and the reviewers. Any product that may be evaluated in this article, or claim that may be made by its manufacturer, is not guaranteed or endorsed by the publisher.

## Footnotes

1
http://www.Arabidopsis.org/

2
http://www.ncbi.nlm.nih.gov/protein

3
http://bioinformatics.psb.ugent.be/webtools/plantcare/html/
