# Supplementary material for: OsQHB Improves Salt Tolerance by Scavenging Reactive Oxygen Species in Rice
Source: Front Plant Sci. 2022 May 4;13:848891. doi: 10.3389/fpls.2022.848891 (PMC9115556; doi:10.3389/fpls.2022.848891)
Supplement: Supplementary file 1 [file Data_Sheet_1.PDF]

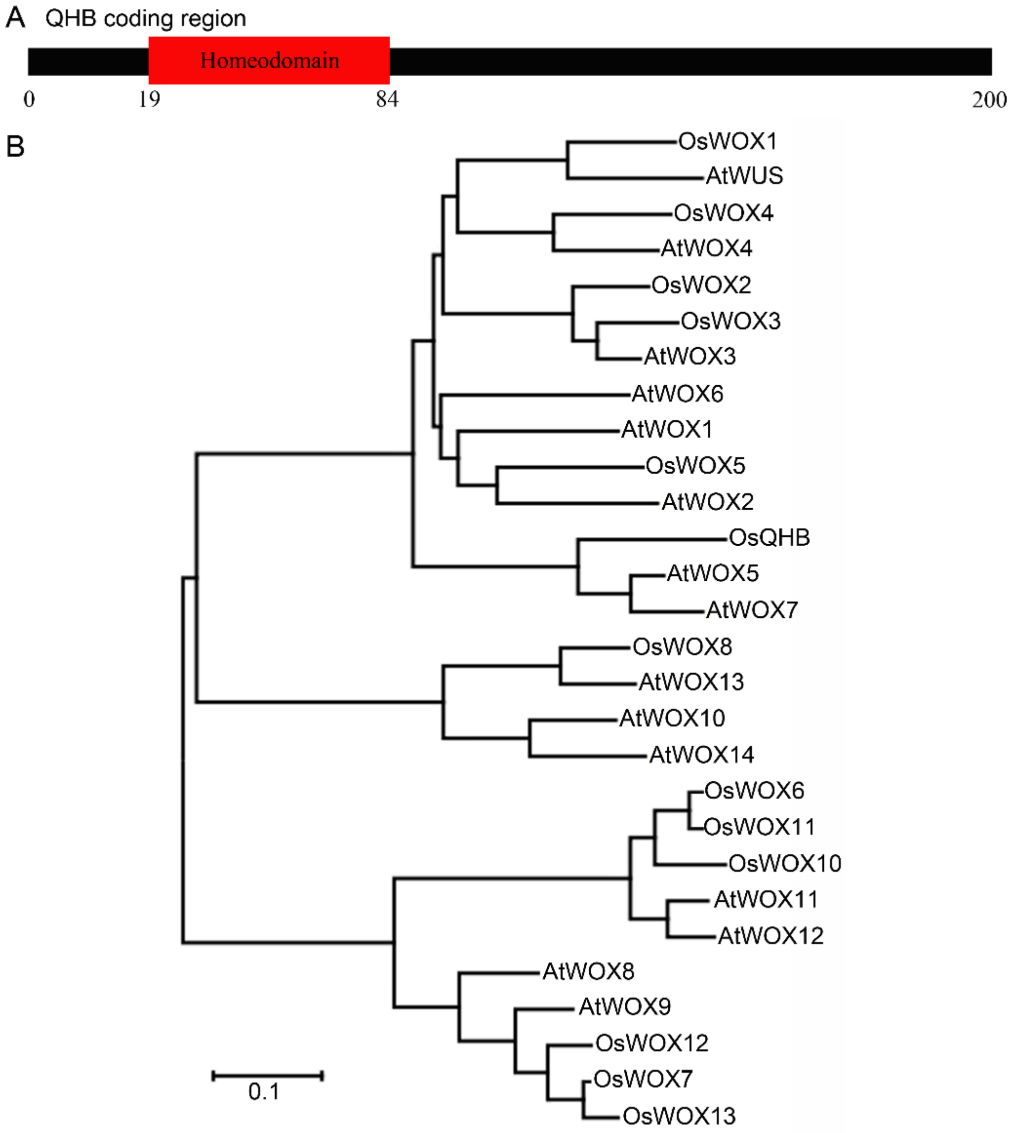

Supplemental Figure 1. Sequence analysis of OsQHB and phylogenetic analysis of OsQHB homologous proteins from rice and *Arabidopsis*.

(A) Schematic diagram of the conserved domains in OsQHB. The conserved homeodomain (HD) domain is marked by red rectangle.

(B) Phylogenetic tree representing evolutionary relationship among WUSCHEL-related homeobox (WOX) family genes from rice and *Arabidopsis*. The amino acid sequences were aligned using the ClustalX 2.0.11 multiple sequence alignment mode, followed by phylogenetic tree construction using MEGA4.0.2.

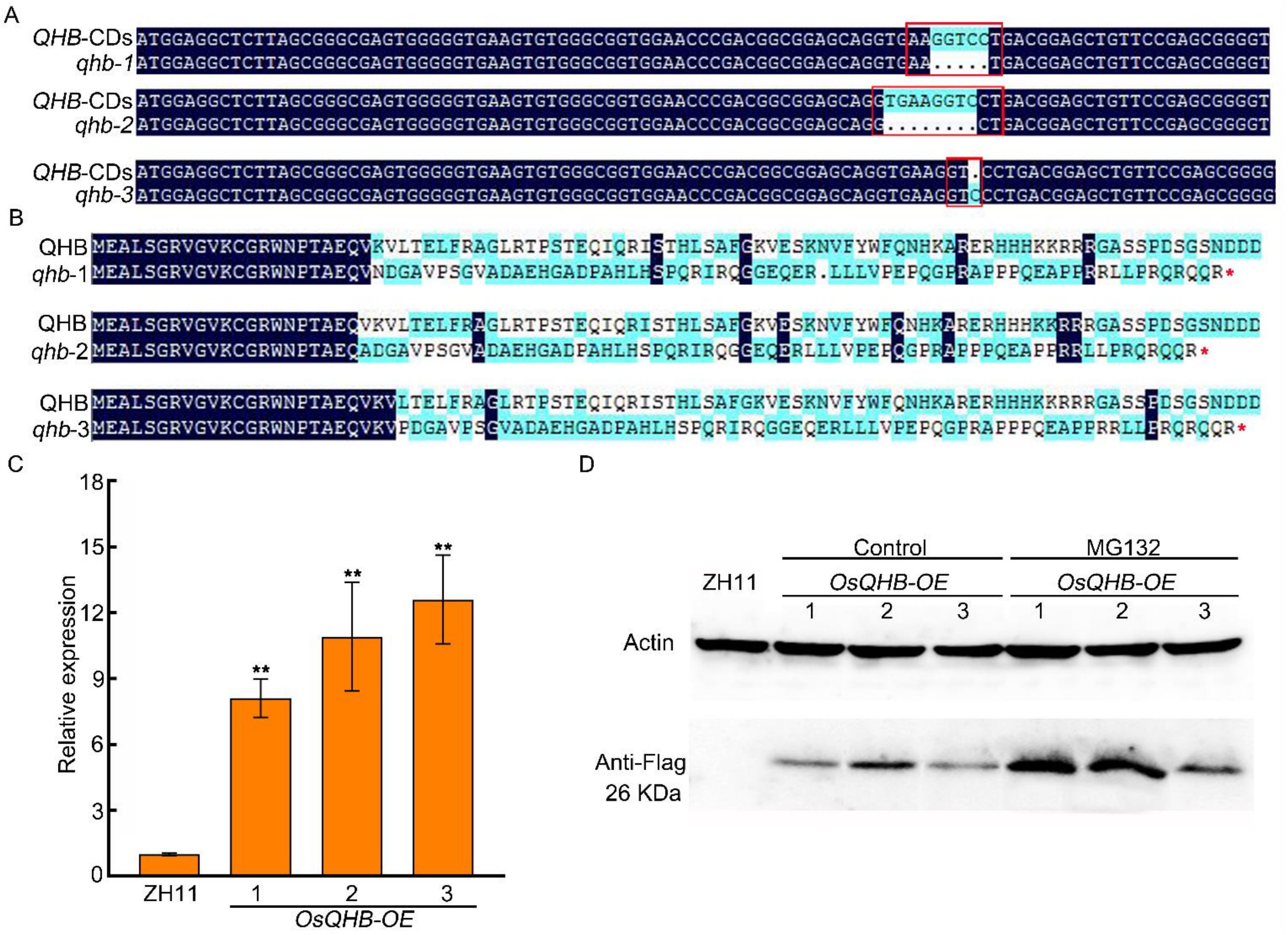

Supplemental Figure 2. Identification of OsQHB mutants and overexpression lines.

(A) DNA sequences of OsQHB in osqhb allelic mutants (osqhb-1, osqhb-2, and osqhb-3) generated by CRISPR/Cas9.

(B) Amino acid sequences of mutants in (A). Both mutations result in a premature protein truncation.

(C) Expression of OsQHB in ZH11 and OsQHB-OE lines. OsActin1 was used as an internal control. The relative expression levels were represented by fold change relative to the expression levels of ZH11. The error bars indicate the SD based on three independent replicates. ** indicates significant difference compared to ZH11 at $P < 0.01$ by Student's t-test.

(D) The level of OsQHB protein in OsQHB-OE plant roots. 3-d-old OsQHB-OE seedlings were treated with or without 2 μm MG132 for 12 h, and total protein was extracted from root and detected by Western Blot.
