# Supplementary material for: OsQHB Improves Salt Tolerance by Scavenging Reactive Oxygen Species in Rice
Source: Front Plant Sci. 2022 May 4;13:848891. doi: 10.3389/fpls.2022.848891 (PMC9115556; doi:10.3389/fpls.2022.848891)
Supplement: Supplementary file 2 [file Table_1.DOCX]

**Supplementary Table S1**: Summary of the AtWOX and OsWOX gene family

| **Gene Name** | **Ensemble Tanscript ID** |
| --- | --- |
| *OsWOX1* | *Os04g0663600* |
| *OsWOX2* | *Os05g0118700* |
| *OsWOX3* | *Os12g0101600* |
| *OsWOX4* | *Os04g0649400* |
| *OsWOX5* | *Os01g0840300* |
| *OsWOX6* | *Os03g0325600* |
| *OsWOX7* | *Os01g0667400* |
| *OsWOX8* | *Os01g0818400* |
| *OsWOX9* | *Os01g0854500* |
| *OsWOX10* | *Os08g0242400* |
| *OsWOX11* | *Os07g0684900* |
| *OsWOX12* | *Os05g0564500* |
| *OsWOX13* | *Os01g0667400* |
| *AtWUSCHEL* | *AT2G17950* |
| *AtWOX1* | *AT3G18010* |
| *AtWOX2* | *AT5G59340* |
| *AtWOX3* | *AT2G28610* |
| *AtWOX4* | *AT1G46480* |
| *AtWOX5* | *AT3G11260* |
| *AtWOX6* | *AT2G01500* |
| *AtWOX7* | *AT5G05770* |
| *AtWOX8* | *AT5G45980* |
| *AtWOX9* | *AT2G33880* |
| *AtWOX10* | *AT1G20710* |
| *AtWOX11* | *AT3G03660* |
| *AtWOX12* | *AT5G17810* |
| *AtWOX13* | *AT4G35550* |
| *AtWOX14* | *AT1G20700* |
