# Supplementary material for: OsQHB Improves Salt Tolerance by Scavenging Reactive Oxygen Species in Rice
Source: Front Plant Sci. 2022 May 4;13:848891. doi: 10.3389/fpls.2022.848891 (PMC9115556; doi:10.3389/fpls.2022.848891)
Supplement: Supplementary file 4 [file Table_3.DOCX]

| **Gene** | **Forward primer (5’-3’)** | **Reverse primer (5’-3’)** |
| --- | --- | --- |
| Primers for qPCR analysis | | |
| *OsActin1* | GACCTTGCTGGGCGTGAT | GTCATAGTCCAGGGCGATGT |
| *OsPRX2* | ACTGCTTTGTCGAGGGTTGT | CGGAGTCGTTGGAGAAGGTC |
| *OsPRX4* | TTGGCACGCATTGATTACCTG | CGATGCGTCGCAACCTTGG |
| *OsPRX9* | TCTTGCTTTCCCTGCTCACC | GCGTCGCATCCCTGAACAA |
| *OsPRX72* | GTGATCGACGAAATCAAGGCG | CCGATGGTGTGGCTACCGAG |
| *OsPRX112* | CAAGGTGAACTCCTCATACGC | GACAGAAGAAGCCGAAGCCA |
| *OsPRX-A2* | GCCGACAATAACCTCCCCAG | AACGTGTGCGCCCCTGAG |
| *OsABSRP5* | CGCCTTCGCCCTGTATGAGAA | CCTCCGTGATCTTGTGCCTG |
| *OsQHB* | TAGCTTTCAAGCAGTTCTACTGC | GAACAGCTCCGTCAGGACCT |
| Primers for CRISPR/Cas9-mediated gene edition of *OsQHB* | | |
| *OsQHB-Cas9* | GGCAGGAGCAGGTGAAGGTCCTGA | AAACTCAGGACCTTCACCTGCTCC |
| Primers for overexpression vector | | |
| *OsQHB-OE* | CCATGGATGGAGGCTCTTAGCGGGCGAG (NcoI) | ACTAGTGAGGCCGAAGCTGCAAAGC (SpeI) |
| Primers for *OsQHB-GFP* vector | | |
| *OsQHB-GFP* | TCCGGAGCTAGCTCTAGAATGGAGGCTCTTAGCGGG | CTTGCTCACCATGGATCCGAGGCCGAAGCTGCAAAG |

**Supplemental Table 3:** Primers used in this study.
